# Potential role of lysine acetylation in the stepwise adaptation of *Candida albicans* to fluconazole

**DOI:** 10.1128/spectrum.02797-24

**Published:** 2025-04-15

**Authors:** Nana Song, Yuying Huang, Xiaowei Zhou, Dongmei Li, Weida Liu, Xiaofang Li

**Affiliations:** 1 Department of Medical Mycology, Hospital for Skin Diseases, Institute of Dermatology, Chinese Academy of Medical Sciences and Peking Union Medical College, Nanjing, Jiangsu, China; 2 Jiangsu Key Laboratory of Molecular Biology for Skin Diseases and STIs, Nanjing, Jiangsu, China; 3 Department of Microbiology & Immunology, Georgetown University Medical Center 12231, Washington, DC, USA; 4 Center for Global Health, School of Public Health, Nanjing Medical University 12461, Nanjing, Jiangsu, China; University of Wisconsin-Madison, Madison, Wisconsin, USA

**Keywords:** *Candida albicans*, acquired fluconazole resistance*in vivo*, lysine acetylation, tandem mass tag labeling

## Abstract

*Candida albicans* is an opportunistic fungal pathogen capable of causing superficial mucosal and systemic infections, sometimes leading to life-threatening conditions. The increasing resistance of *C. albicans* to azole antifungals has become a significant challenge in clinical treatment. Lysine acetylation (KAc) is a well-studied post-translational modification that plays crucial roles in various biological processes. However, its impact on antifungal resistance in *C. albicans* remains poorly understood. Five strains of *C. albicans* isolated from the same patient, representing different stages of acquired fluconazole resistance *in vivo*, were used in this study to investigate the potential regulatory mechanism of KAc on the development of azole resistance in *C. albicans*. Quantitative proteomic analysis using tandem mass tag (TMT) labeling, acetylation enrichment, and liquid chromatography-mass spectrometry (LC-MS) was conducted on these five strains. We divided all strains into four comparison groups and identified a total of 1,796 lysine acetylation sites across 938 proteins, with quantitative data available for 1,314 acetylation sites in 712 proteins. Analysis of 155 significantly differentially modified sites revealed that the acetylation levels of key proteins involved in the conversion of pyruvate to acetyl-CoA for entry into the tricarboxylic acid (TCA) cycle for energy production were initially decreased and then increased during the acquisition of fluconazole resistance. Additionally, the acetylation levels of proteins involved in ribosome synthesis, translation processes, and amino acid synthesis were found to increase. Therefore, lysine acetylation in *C. albicans* may contribute to azole resistance by regulating energy metabolism and protein synthesis.

**I**MPORTANCE**:**

*Candida albicans*, an opportunistic fungal pathogen, presents significant clinical challenges due to its escalating resistance to azole antifungals, especially fluconazole. This study investigates the role of lysine acetylation in the development of azole resistance using multiple strains isolated from a single patient with varying resistance levels. Through advanced proteomic analysis, we identified numerous lysine acetylation sites on proteins involved in key metabolic pathways. The results revealed a dynamic change in the acetylation of proteins related to energy metabolism — specifically, those connecting pyruvate to the tricarboxylic acid cycle—which correlated with the evolution of resistance. Additionally, increased acetylation was observed in proteins linked to ribosome synthesis and translation processes. These findings suggest that lysine acetylation is crucial for regulating metabolic and protein synthesis pathways, potentially influencing azole resistance in *C. albicans.*

## INTRODUCTION

Globally, an estimated 250,000 patients suffer from invasive candidiasis each year, resulting in over 50,000 deaths (1). *Candida albicans* remains the leading fungal pathogen, responsible for 90%–100% of superficial mucosal infections and 40%–70% of disseminated infections (2). According to a survey conducted by the Transplant-Associated Infection Surveillance Network in the United States, *C. albicans* accounted for 46.3% of invasive candidiasis cases (3). Similarly, in critically ill patients in Asian countries like India, China, and Thailand, 43.1% of *Candida* bloodstream infections were attributed to *C. albicans* (4).

Fluconazole (FLC) is one of the first-line antifungals commonly used to treat *C. albicans* infections due to its broad antifungal spectrum, low toxicity, and high bioavailability (5). However, as a fungistatic drug, FLC is prone to inducing resistance, especially during prophylaxis, empiric therapy, irregular treatment, and prolonged treatment courses. The rising incidence of azole resistance in *C. albicans* has become a significant clinical concern.

Current research on drug resistance mechanisms in *C. albicans* primarily focuses on genetic mechanisms, including mutations or overexpression of the target enzyme coding gene (*ERG11*), increased expression of drug efflux pump-related genes (*CDR1*, *CDR2*, and *MDR1*), biofilm formation, cellular metabolic stress responses, and mutations in drug resistance-related transcription factors, especially zinc cluster transcription factors such as *TAC1*, *MRR1*, and *UPC2* (6). In addition, large-scale genomic alterations, including aneuploidy, loss-of-heterozygosity (LOH), and chromosomal rearrangements, can affect the expression of drug targets, efflux pumps, and other resistance factors (7). The development of azole resistance in *C. albicans* is not regulated by a single mechanism but involves multiple mechanisms that collectively lead to high levels of resistance over time.

However, similar to other biological processes, data from genomic or transcriptomic analyses may not accurately reflect the actual protein functions within cells. In contrast to transcriptomics, proteomics provides a more direct connection to protein function and phenotype (8, 9). Precise regulation of protein function is essential for various biological processes, including drug resistance.

Post-translational modifications (PTMs) can modulate protein function more rapidly and with lower energy costs compared to protein turnover (10). Lysine acetylation is an evolutionarily conserved PTM found in both eukaryotic and prokaryotic organisms. It is characterized by the addition or removal of acetyl groups from lysine residues, a process mediated by lysine acetyltransferases (KATs) and lysine deacetylases (KDACs) (10–12). It has been proposed that KATs and KDACs interact closely with specific transcriptional regulators to establish a dual-layer network for chromatin-mediated transcriptional regulation in *C. albicans* (13). Chromatin modification factors, including KATs such as *GCN5*, *HAT1*, and *RTT109*, as well as KDACs like *SET3*, *HAD1, RPD31,* and *HST3*, are implicated in the virulence processes of *Candida* species, including filamentous growth and biofilm formation, as well as in their resistance and sensitivity to antifungal drugs (14–16). Moreover, many small-molecule inhibitors targeting KDACs have been identified, with several undergoing clinical trials as potential anticancer agents. In *Saccharomyces cerevisiae* and *C. albicans*, the broad-spectrum KDAC inhibitor Trichostatin A (TSA) has been shown to reverse azole resistance (17). Additionally, the KDAC inhibitor MGCD290, targeting the fungal *HOS2* complex, demonstrates synergistic effects when combined with azoles and echinocandins on various drug-resistant isolates of *C. albicans* (18, 19). In a previous work, we identified 477 acetylated proteins (5.28%) out of 9,038 proteins in *C. albicans* (20), providing a crucial foundation for further functional analysis of acetylated proteins in *C. albicans*. Subsequently, lysine acetylation has also been identified in *Candida glabrata* (21), *Cryptococcus neoformans* (22), *Trichophyton rubrum* (23), and *Aspergillus fumigatus* (24), highlighting its broader significance in fungal biology.

Here, we utilized tandem mass tag (TMT) labeling analysis based on normalized proteomic data (25) to compare lysine acetylation levels in *C. albicans* strains isolated before and after FLC treatment. The strains analyzed included Ca1 (MIC = 0.25 µg/mL, sensitive), Ca2 (MIC = 1 µg/mL, sensitive), Ca8 (MIC = 8 µg/mL, initial resistance), Ca14 (MIC = 32 µg/mL, increased resistance), and Ca17 (MIC >64 µg/mL, highly resistant). All five strains were sourced from Professor T. C. White (School of Biological Sciences, University of Missouri at Kansas City, Kansas City, MO, USA) and were isolated from an AIDS patient who experienced recurrent oropharyngeal candidiasis over a 2-year period (26). In this collection, FLC resistance was thought to develop gradually *in vivo* and was associated with the dosages administered to the patient. Molecular analyses confirmed that these isolates are derived from a single *C. albicans* strain, with their resistance levels remaining stable across 600 generations (27). Over the past 20 years, these strains have been extensively investigated at the genomic and transcriptomic levels to elucidate the mechanisms of resistance (25–35), revealing a combination of various resistance mechanisms among the resistant strains (Table 1). This research represents the first comprehensive examination of lysine acetylation changes associated with the progressive acquisition of FLC resistance, providing new insights into strategies for combating this pathogenic fungus.

**TABLE 1:** Antifungal profiles and resistance of strains in this study^*b*^

Strain (27)	FLC (mg/d)	MIC (μg/mL) (35)						Previously described resistance mechanism(s) (25)
		FLC^*a*^	FLC	ITR	VOR	AMB	CAS	
Ca1	100	0.25	0.5	0.031	0.0313	0.25	0.0313	/^*c*^
Ca2	100	1	2	0.031	0.0313	0.25	0.0625	GOF mutations in *MRR1*; transient LOH events occurred on chromosome R; overexpression of *MDR1*
Ca8	100	8	8	0.031	0.0313	0.5	0.0625	Transient ploidy changes
Ca14	400	32	32	0.063	0.125	0.25	0.125	Overexpression of *ERG11*; loss of allelic variation in *ERG11*; *R467K* mutation in *ERG1*
Ca17	800	>64	128	0.5	1	0.5	0.125	GOF mutations in *MRR1*, *TAC1*, and *UPC2*; mitotic recombination on chromosome 5 left arm; overexpression of *ERG11*, *MDR1*, *CDR1*, and *CDR2*; loss of allelic variation in *ERG11*; *R467K* mutation in *ERG11*

^*a*^ This MIC value of FLC was measured by White et al. in 1997 (28).

^*b*^ FLC, fluconazole; ITR, itraconazole; VOR, voriconazole; AMB, amphotericin B; CAS, caspofungin.

^*c*^ /, none.

## MATERIALS AND METHODS

Five *C. albicans* isolates were initially cultured overnight at 28°C in yeast extract peptone dextrose (YPD) medium with continuous shaking at 220 rpm to prepare seed cultures. The overnight cultures were diluted with fresh YPD liquid medium to an initial OD_600_ of 0.1, then incubated at 28°C for 4 hours with 220 rpm shaking until the OD_600_ reached 0.8. The cultured cells were collected by centrifugation at 6,000 rpm for 10 minutes at 4°C and washed twice with cold phosphate-buffered saline. Each treatment was carried out in triplicate. The methods for protein extraction, trypsin digestion, TMT labeling, HPLC fractionation, LC-MS/MS analysis, and database searching have been previously described without modification (25).

### Enrichment of acetylated peptides

To enrich acetylated peptides, the enzymatically digested peptides were dissolved in IP buffer (100 mM NaCl, 1 mM EDTA, 50 mM Tris-HCl, 0.5% NP-40, pH 8.0) and then co-incubated with pre-washed acetylation resin (catalog number PTM-104, PTM Bio Lab, Inc.) by slow shaking at 4°C overnight. After co-incubation, the peptides were washed four times with IP buffer and twice with ddH_2_O. Subsequently, the bound peptides were eluted from the antibody beads thrice with 0.1% trifluoroacetic acid. Finally, the eluted fractions were collected and vacuum-dried. The peptides were then desalted using C18 ZipTips (Millipore) according to the manufacturer’s instructions for LC-MS/MS analysis.

### Bioinformatics data analysis

We used Motif-x to identify peptide motifs surrounding lysine acetylation sites in protein sequences, examining 10 amino acids upstream and downstream of each site. Database searches used default parameters. Motifs were defined as sequences present in >20 peptides with a *P*-value < 0.000001.

The cluster analysis based on the functional enrichment (Gene Ontology: molecular function, cellular component, and biological process; KEGG pathway; and protein domain) of proteins with differentially abundant lysine acetylation sites and the dynamic cluster analysis were similar to the methods described previously (25).

### Parallel reaction monitoring

Acetylation-targeted quantification through parallel reaction monitoring (PRM) is a mass spectrometry-based technique that does not rely on antibodies and allows simultaneous verification of multiple target proteins or modification sites with high sensitivity and accuracy (36). The peptides were dissolved in mobile phase A (0.1% formic acid and 2% acetonitrile in water) and separated using an EASY-nLC 1200 (Thermo Fisher) ultra-HPLC system. The liquid gradient was set as follows: 0–36 min, 9%–25% mobile phase B (0.1% formic acid and 90% acetonitrile in water); 36–52 min, 25%–35% B; 52–56 min, 35%–80% B; 56–60 min, 80% B, with a flow rate maintained at 500 nL/min.

Peptides were separated using the ultra-HPLC system, ionized using an NSI source, and then analyzed by Q Exactive HF-X mass spectrometry. The source voltage was set to 2.1 kV. Peptide precursor ions and fragment ions were detected and analyzed using a high-resolution Orbitrap. The MS1 scan range was set to 390–1,020 *m*/*z* with a resolution of 120,000, while the MS2 scan resolution was set at 15,000. In data-dependent acquisition mode, the high energy collision dissociation (HCD) fragmentation energy was set to 28. The automatic gain control (AGC) for MS1 was set to 3E6 with a maximum injection time of 50 ms. For MS2, the AGC was set to 1E5 with a maximum injection time of 200 ms, and an isolation window of 1.4 *m*/*z* was used. Data were processed using Skyline 20.2 software.

## RESULTS

### Comparative acetylome analysis of serial *C. albicans*

In total, 1,796 lysine acetylation (KAc) sites in 938 proteins were identified across 4 *C. albicans* groups (Q1 = Ca2 vs Ca1, Q2 = Ca8 vs Ca1, Q3 = Ca14 vs Ca1, Q4 = Ca17 vs Ca1). Among these, 1,314 KAc sites in 712 proteins were quantitated (Data set S1). Notably, no quantitative KAc sites were detected in the known efflux pump proteins listed in Table 1 (such as Mdr1, Cdr1, and Cdr2). Although multiple KAc sites were identified in the steroid biosynthesis pathway proteins (Erg10, Erg11, Erg12, Erg13, Erg251, Erg3, Erg5, Erg6, and Erg9), the levels at these KAc sites did not show significant changes across the four comparative groups (Q1–Q4). Based on the criteria of *P* < 0.05 and a fold change >1.5 or <0.667 in at least three biological replicates, 155 KAc sites with significant changes and their corresponding 123 differentially acetylated proteins (DAPs) were identified (Data set S2) and summarized in Table S1. The DAPs were primarily involved in mitochondrial respiration, carbohydrate metabolism, amino acid synthesis metabolism, lipid degradation, DNA repair, histone modification, ribosome synthesis and protein translation, and transport.

As depicted in Table 2, more acetylated sites and proteins showed increased acetylation levels in Q2 (31 KAc sites in 28 proteins) and Q3 (48 KAc sites in 38 proteins) compared to Q1, while fewer acetylation sites and proteins were identified in Q1 (16 KAc sites in 14 proteins) and Q4 (21 KAc sites in 20 proteins). Moreover, 43, 37, 6, and 36 KAc sites significantly showed decreased abundance from 36, 31, 6, and 31 proteins in four comparison groups.

**TABLE 2:** Differentially expressed KAc sites and modified proteins

Groups	Increased (>1.5)		Decreased (<0.667)	
	KAc sites	Proteins	KAc sites	Proteins
Q1 (Ca2 vs Ca1)	16	14	43	36
Q2 (Ca8 vs Ca1)	31	28	37	31
Q3 (Ca14 vs Ca1)	48	38	6	6
Q4 (Ca17 vs Ca1)	21	20	36	31

Moreover, 8 core histones with 44 KAc sites were found to have altered acetylation levels. Among these, 8 KAc sites in 7 core histones (Histone H4, Histone H3.1/H3.2, Histone H2A.2, Histone H2B.2, Histone H2B.2, and Histone H2A.Z) showed significant changes in acetylation (Table 3). Additionally, one lysine acetyltransferases, ESA1, exhibited increases in acetylation at two specific sites, ESA1K123Ac and ESA1K127Ac. These acetylome data suggest that a substantial number of proteins alter their acetylation pattern during the development of drug resistance in *C. albicans*.

**TABLE 3:** Differentially expressed KAc sites in seven core histones

Protein accession	KAc site	Protein description	Gene name	Ratio			
				Q1	Q2	Q3	Q4
Q59VN4	19	Histone H4	*HHF1*	0.737	0.635	1.043	0.922
A0A1D8PR93	165	Histone H1	*HHO1*	1.334	1.613	0.83	0.994
Q59VN2	56	Histone H3.1/H3.2	*HHT21*	1.798	1.563	1.164	1.154
Q59VN2	79	Histone H3.1/H3.2	*HHT21*	0.738	0.574	1.037	0.663
Q59VP2	5	Histone H2A.2	*HTA2*	0.701	0.569	0.879	0.89
Q59VP1	22	Histone H2B.2	*HTB2*	0.857	0.666	0.856	0.776
Q59VP1	23	Histone H2B.2	*HTB2*	0.869	0.589	0.877	0.747
Q5AEE1	4	Histone H2A.Z	*HTZ1*	0.93	0.594	1.063	1.213

### Motif analysis of acetylated proteins

To characterize the specific sequence motifs surrounding acetylated lysine residues, we generated a sequence logo (Fig. S1). Sixteen significantly enriched motifs were identified including KAc*E, KAcF, LKAcK, KAcH, KKAcS, A*KAc*A, KAc*D, FKAcK, KAcN, KAcK, KAcS, KAcT, KAcR, K****KAc, K***KAc, A*******KAc (where KAc represents the acetylated lysine and * represents a random amino acid residue, Fig. S1A). To determine if specific amino acids are adjacent to acetylated lysines, we analyzed the amino acid sequences flanking these sites using a heat map (Fig. S1B). Alanine (A), lysine (K), and arginine (R) were expressed in multiple positions surrounding KAc, suggesting their crucial role in sequence motifs.

### Enrichment-based cluster analysis

To better understand the functional characteristics of DAPs during the acquired resistance process of *C. albicans*, we performed clustering analyses based on enrichment of GO classification (Fig. S2-S5), KEGG pathway (Fig. S6), and protein domain (Fig. S7). These analyses provide an intuitive view of the functional changes of DAPs across different comparison groups by visualizing the enrichment results on a heatmap.

### Cluster analysis based on GO enrichment

**Fig 1:**
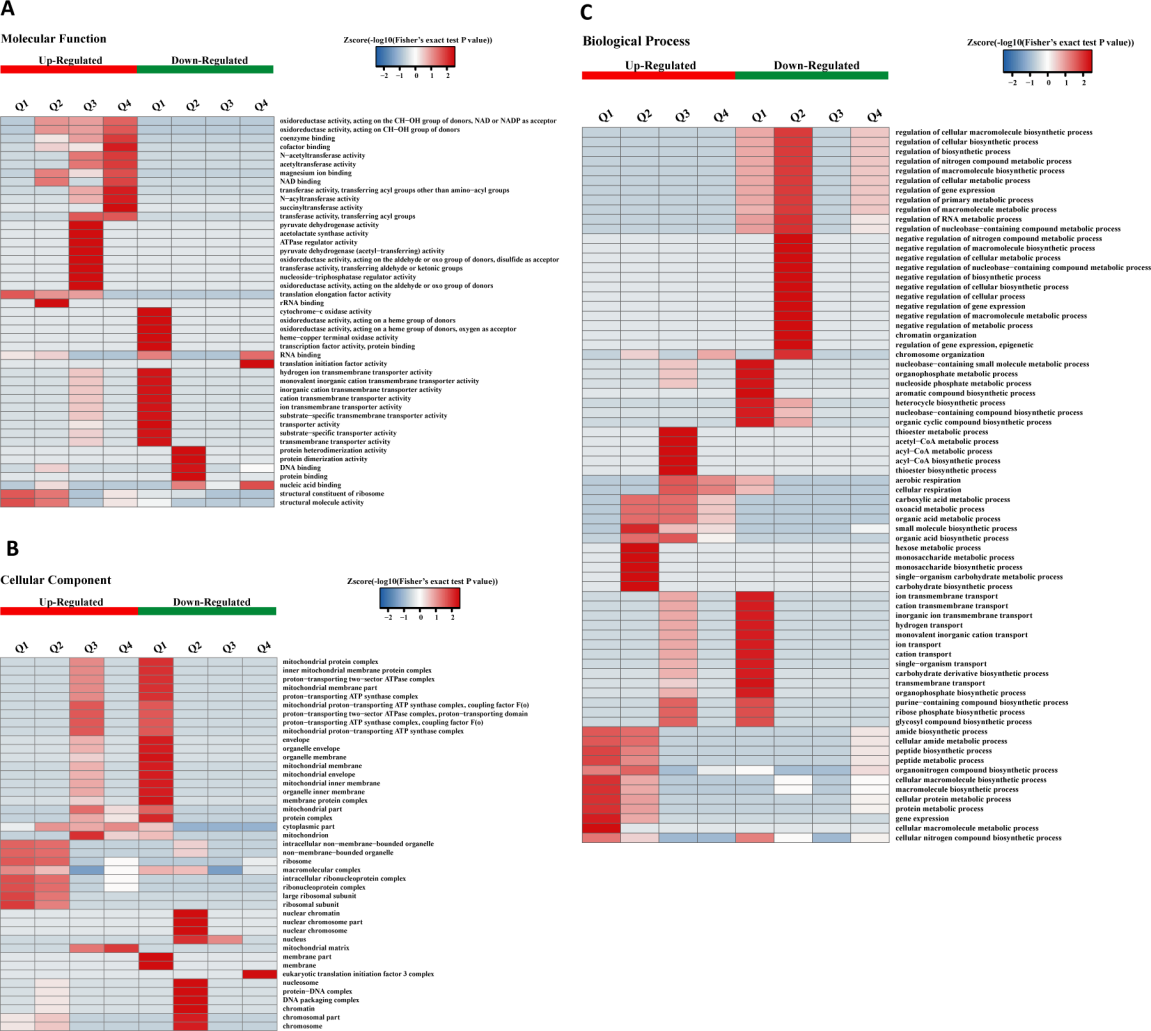
Hierarchical clustering analysis was conducted for DAPs according to GO-based enrichment including molecular function (**A**), cellular component (**B**), and biological process (**C**). The *P* values were transformed into *Z*-scores for hierarchical clustering analysis. The *Z*-score is shown in the color legend, and the red color represents significant enrichments.

As shown in Fig. 1A through C, GO enrichment-based clustering analysis of the DAPs was performed across three categories: molecular functions, cellular components, and biological processes. In Q1, the DAPs with increased acetylation levels were primarily enriched in ribosomal structural constituents, structural molecular activity, and translation elongation factor activity. These proteins are mainly associated with cellular components like non-membrane-bound organelles, ribosomes, and macromolecular complexes. They participate in biological processes such as peptide biosynthesis, translation, and peptide metabolism. Conversely, the DAPs with decreased acetylation levels in Q1 were mainly enriched in monovalent inorganic cation transmembrane transporter activity, hydrogen ion transmembrane transporter activity, and inorganic cation transmembrane transporter activity. These DAPs were primarily located in cellular components such as membrane protein complexes, proton-transporting ATP synthase complexes, and mitochondrial inner membrane protein complexes. They are involved in processes like monovalent inorganic cation transmembrane transport, hydrogen ion transmembrane transport, and transport processes.

In Q2, the proteins exhibiting increased KAc levels were mainly involved in ribosomal structural constituents, organic nitrogen compound metabolic processes, amide biosynthetic processes, translation, and monosaccharide and hexose metabolic processes. These DAPs are also concentrated in cellular components such as non-membrane-bound organelles, ribosomes, and macromolecular complexes. Conversely, the DAPs with decreased KAc levels in Q2 were primarily enriched in cellular components like nucleoli, protein-DNA complexes, and chromatin. Their molecular functions include protein homodimer activity, DNA binding, and chromatin silencing. These proteins participate in biological processes such as the negative regulation of gene expression, chromatin silencing, and the negative regulation of RNA template transcription.

In Q3, the DAPs with enhanced acetylation were mainly associated with transferase activity, acetyl lactate dehydrogenase activity, pyruvate dehydrogenase activity, and adenosine triphosphatase regulatory activity. These proteins are involved in processes such as energy-coupled proton transport, ATP biosynthesis, and branched-chain amino acid synthesis metabolism. The key cellular component where they function is the mitochondria.

In Q4, the hyperacetylated DAPs are mainly related to acyltransferase activity, excluding aminoacyl transferase activity, succinyl-transferase activity, and NAD binding activity. These proteins participate in processes related to the tricarboxylic acid cycle metabolism, and aerobic respiration, with the associated DAPs primarily localized in the cytoplasm and mitochondria. The DAPs showing decreased acetylation in Q4 were mainly enriched in nucleic acid binding, translation initiation, and organic nitrogen compound biosynthetic processes.

### Cluster analysis based on KEGG pathway enrichment

**Fig 2:**
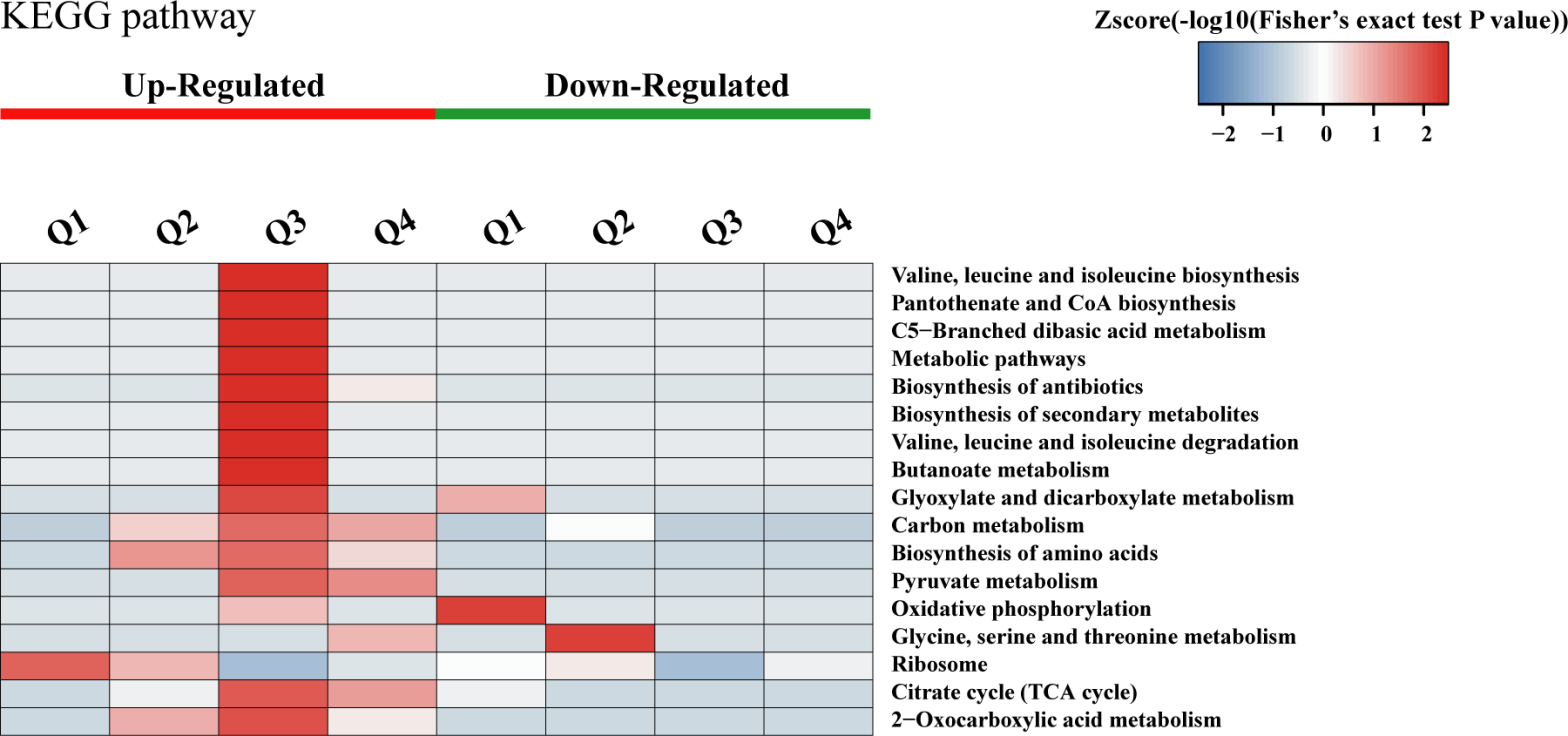
Hierarchical clustering analysis was conducted for DAPs according to KEGG pathway-based enrichment. The *P* values were transformed into *Z*-scores for hierarchical clustering analysis. The *Z*-score is shown in the color legend, and the red color represents significant enrichments.

To elucidate the characteristic changes in metabolic pathways of DAPs during the acquired resistance process, we conducted a cluster analysis based on the KEGG pathway enrichment of DAPs (Fig. 2). For Q1, the DAPs showing increased KAc levels were significantly enriched in the ribosome pathway, while DAPs with decreased abundance were mainly involved in oxidative phosphorylation. In Q2, pathways related to 2-oxocarboxylic acid metabolism, ribosome, and amino acid biosynthesis were enriched with DAPs present at elevated levels. Meanwhile, theglycine, serine, and threonine metabolism and the ribosome pathway were primarily associated with DAPs whose abundance was diminished. In Q3, DAPs with enhanced KAc levels showed significant enrichment in several pathways, including the tricarboxylic acid (TCA) cycle, 2-oxocarboxylic acid metabolism, antibiotic biosynthesis, valine, leucine, and isoleucine biosynthesis and degradation, pantothenate and CoA biosynthesis, carbon metabolism, pyruvate metabolism, and amino acid biosynthesis pathways. In Q4, the TCA cycle, 2-oxocarboxylic acid metabolism, pyruvate metabolism, and carbon metabolism were primarily linked to DAPs showing elevated levels. No enriched pathways were identified for DAPs with decreased presence in Q3 and Q4.

### Cluster analysis based on protein domain enrichment

**Fig 3:**
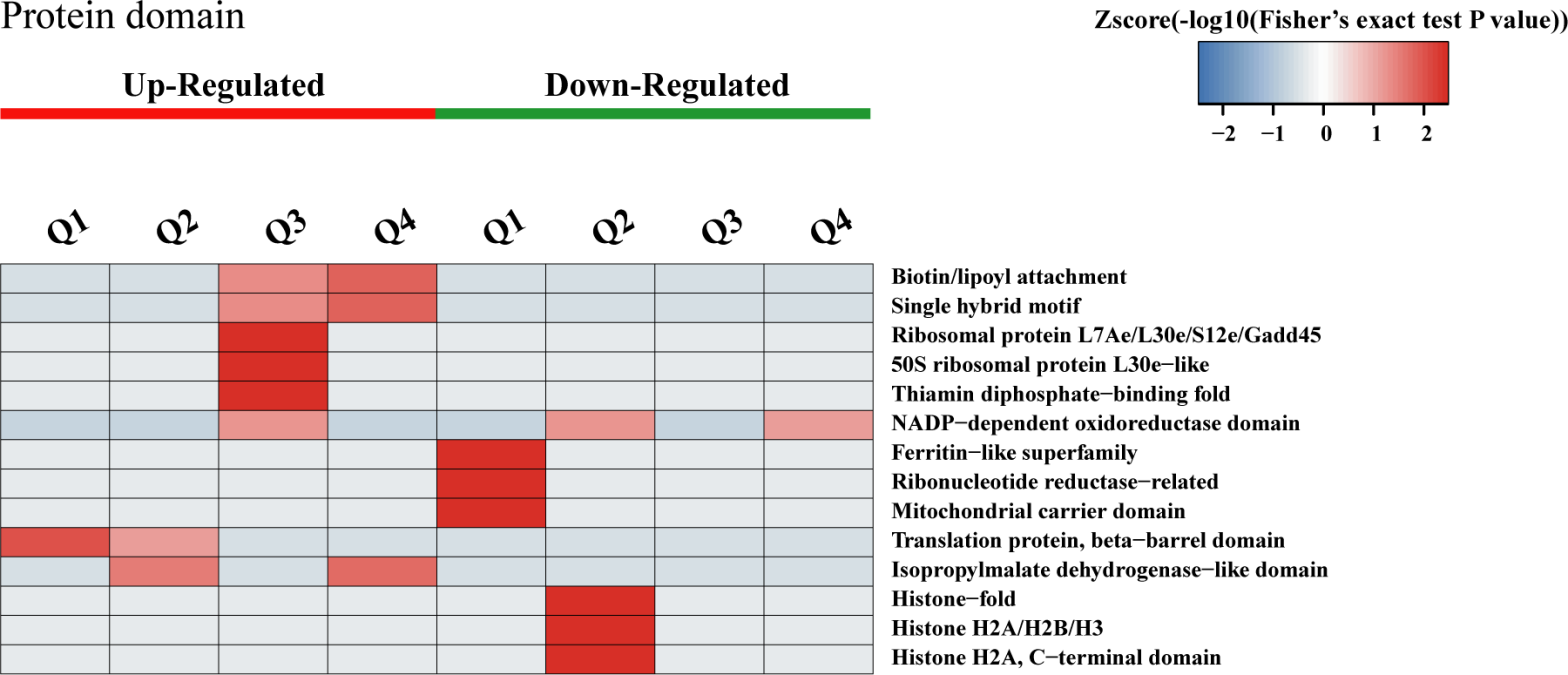
Hierarchical clustering analysis was conducted for DAPs according to protein domain-based enrichment. The *P* values were transformed into *Z*-scores for hierarchical clustering analysis. The *Z*-score is shown in the color legend, and the red color represents significant enrichments. The *P* values were transformed into *Z*-scores for hierarchical clustering analysis. The *Z*-score is shown in the color legend, and the red color represents significant enrichments.

Protein domains are conserved regions of proteins that can function independently of the rest of the protein. To explore the structural domain characteristics of DAPs during the acquired resistance process, we conducted cluster analysis based on protein domain enrichment (Fig. 3). In Q1, DAPs showing increased abundance were primarily enriched in translation protein beta-barrel domains, while DAPs with lower levels were mainly associated with ferritin-like superfamily, ribonucleotide reductase, and mitochondrial carrier domains. In Q2, the more abundant DAPs were mainly enriched in isopropyl malate dehydrogenase-like domains and translation protein beta-barrel domains. However, DAPs with reduced KAc levels were largely related to histone folding, histone H2A/H2B/H3, and NADP-dependent oxidoreductase domains. For Q3, DAPs exhibiting higher KAc levels were mainly enriched in thiamine pyrophosphate-binding fold, ribosomal protein L7Ae/L30e/S12e/Gadd45, 50S ribosomal protein L30e-like, single hybrid motif, and biotin/lipoyl attachment domains. In Q4, DAPs showing increased levels were primarily enriched in isopropyl malate dehydrogenase-like domains, single hybrid motif, and biotin/lipoyl attachment domains, whereas DAPs with decreased KAc levels were mainly enriched in NADP-dependent oxidoreductase domains.

### Dynamic cluster analysis of expression patterns

**Fig 4:**
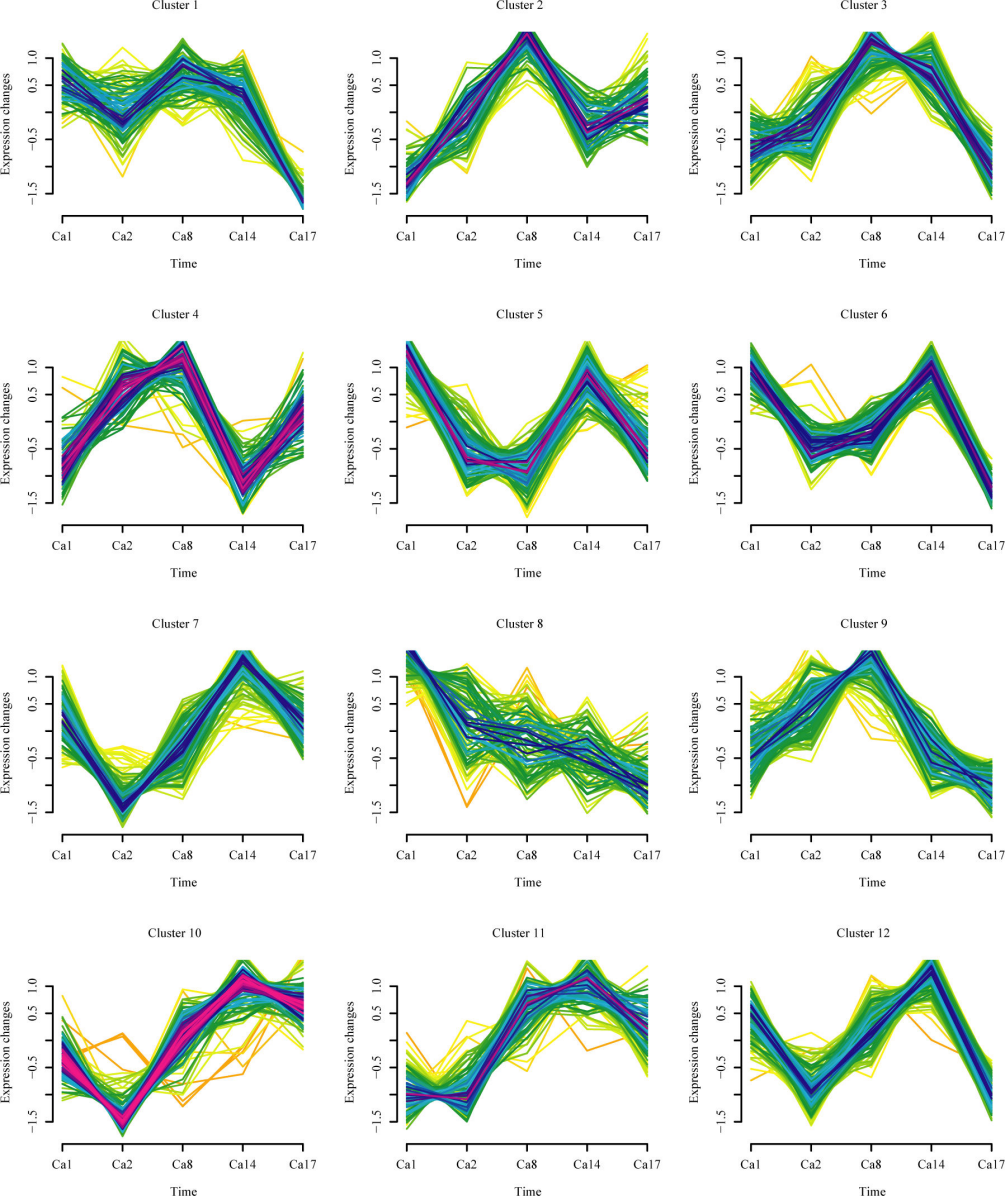
Dynamic clustering of 1,171 quantitative KAc sites, classified into 12 clusters. Clusters 4, 5, and 10 account for a significant proportion of the differentially expressed KAc sites.

Quantitative data for 1,314 acetylation sites in 712 proteins were obtained in this study. Based on the trends in acetylation site abundance across the four comparative groups, 1,171 quantitative KAc sites were classified into 12 clusters (Fig. 4 and Table S2). Notably, none of the 155 significantly increased and decreased KAc sites were classified into Cluster 1, while the remaining 11 clusters (Clusters 2–12) contained differential KAc sites in the following numbers: 9, 2, 17, 39, 9, 13, 13, 5, 32, 15, and 1. Cluster 4, Cluster 5, and Cluster 10 had a higher proportion of differential KAc sites, demonstrating significant attenuation in Ca14, Ca8, and Ca2, respectively. In Cluster 4, all differential KAc sites showed increased acetylation levels, and the corresponding DAPs were mainly associated with the ribosomal large subunit, hexokinase, fatty acid synthase subunit, and aspartate synthase subunit. Conversely, in Cluster 5, all KAc sites exhibited decreased acetylation levels, and the corresponding DAPs were primarily involved in histone modification and ribosome synthesis. Meanwhile, the differential KAc sites in Cluster 10 displayed a decrease in acetylation levels at Ca2, followed by a gradual increase from Ca8 to Ca17. The corresponding DAPs in this cluster were mainly involved in mitochondrial respiration and the tricarboxylic acid cycle.

### PRM-based validation

To further validate our acetylome results, eight differential KAc sites were selected for PRM assay based on their functional significance and parameters derived from the omics data. As shown in Table 4, the PRM results closely align with the omics data, which confirmed the reliability of the whole-cell acetylome findings. Notably, H3K79ac displayed the most pronounced reduction in abundance in strain Ca8, where DAPs exhibiting reduced abundance were significantly enriched in molecular functions such as negative regulation of gene expression, chromatin silencing, and gene silence.

**TABLE 4:** PRM results of eight KAc sites among four comparison groups

Gene name	KAc site	Protein description	Ratio (KAc)				Ratio (PRM)			
			Q1	Q2	Q3	Q4	Q1	Q2	Q3	Q4
*CAALFM _CR04450CA*	393	Chromatin modification protein	0.57	0.66	0.65	0.65	0.79	0.56	0.81	0.60
*DPM1*	170	Dolichyl-phosphate beta-D-mannosyltransferase	0.68	0.15	0.40	0.40	0.74	0.57	1.11	0.58
*FHL1*	775	Fork-head transcriptional regulator	0.87	0.84	0.80	0.80	0.62	1.18	0.88	0.73
*HHT21*	79	Histone H3.1/H3.2	0.66	0.52	0.56	0.56	0.64	0.27	0.86	0.55
*IDH1*	298	Isocitrate dehydrogenase subunit	1.21	1.91	1.80	1.80	1.32	1.29	1.46	1.50
*RPL21A*	97	Ribosomal 60S subunit protein L21A	2.32	3.29	2.49	2.49	1.43	1.26	1.53	1.78
*RPL82*	15	Ribosomal 60S subunit protein L82	1.08	1.14	1.35	1.35	0.84	1.17	1.40	1.56
*SAC6*	135	Fimbrin	0.43	0.38	0.49	0.49	0.64	0.54	0.88	0.70

## DISCUSSION

Lysine acetylation is a crucial and highly conserved PTM with diverse biological functions in both eukaryotes and prokaryotes. This study represents the first comprehensive investigation into the dynamic changes in lysine acetylation levels in *C. albicans* during the acquisition of FLC resistance *in vivo*. Based on our quantitative data, neither significant changes in the acetylation levels of sterol-synthesis proteins nor quantitative information on acetylation sites of export pumps were found. Therefore, we speculate that acetylation changes may not directly regulate the acetylation levels of these proteins to influence the acquired resistance mechanism. However, they might still affect drug resistance indirectly through other pathways.

Previous studies have shown that most identified acetylated proteins are involved in the regulation of glucose, lipid, and amino acid metabolism (37, 38). Reversible acetylation of metabolic enzymes enables cells to rapidly sense their energy status and flexibly adapt their metabolic rates or directions in response to environmental changes (39). In this study, we observed significant alterations in the acetylation levels of proteins involved in metabolism. Key enzymes involved in the acetyl-CoA to the tricarboxylic acid cycle, such as Pda1, Pdb1, Pdx1, Lpd1, Cit1, Kgd2, and Lsc2, exhibited decreased acetylation levels in the sensitive strain Ca2, followed by an increase in Ca8, Ca14, and Ca17. Notably, citrate synthase Cit1 exhibited significant differential expression at its acetylation modification sites K48, K190, K450, K148, and K102. Similarly, the acetylation levels of eight sites in six subunits of mitochondrial F1FO ATP synthase showed a pattern of decrease followed by an increase, peaking in the Ca14 strain. These subunits include the β subunit (Atp2), b subunit (Atp4), OSCP subunit (Atp5), d subunit (Atp7), f subunit (Atp17), and g subunit (Atp20). These findings highlight that acetylation modifications in *C. albicans* are involved in regulating drug resistance-related energy metabolism.

Inactivation of the TCA cycle or energy-generating metabolism is a common strategy for bacteria to develop antibiotic resistance or tolerance. Increased acetylation levels of central metabolism-related proteins can negatively regulate bacterial energy metabolism, reducing metabolic activity and modulating antibiotic resistance (40, 41). For example, deacetylation of the K413 site of pyruvate kinase in *Escherichia coli* enhances enzyme activity, leading to increased bacterial energy metabolism and restored sensitivity of antibiotic-resistant strains to ampicillin, kanamycin, and polymyxin B (42). In eukaryotic cells, evolutionarily conserved lysine acetylation modifications in the α, β, γ, and OSCP subunits of ATP synthase can be reversed by the deacetylase Sirt3 (43). Acetylation of these subunits is associated with decreased enzyme activity (44). Therefore, it is speculated that under prolonged FLC exposure, *C. albicans* adapts to the environment by reducing its metabolic levels through acetylation, thereby acquiring drug resistance.

In contrast to the decreased acetylation observed in energy metabolic enzymes, the acetylation levels of proteins involved in ribosome synthesis, translation processes, peptide synthesis, and amino acids (leucine, valine, isoleucine, lysine, arginine, aspartate, and glutamine) synthesis were elevated. This increase aligns with the reported rise in ribosomal subunit abundance in antibiotic resistance studies (45). Therefore, we hypothesized that *C. albicans* may also regulate ribosome synthesis and translation activity through acetylation, enhancing its growth and resistance to external FLC pressure. In conclusion, the dynamic interplay between cellular metabolism and epigenetic mechanisms, particularly through acetylation, may play a crucial role in the development of drug resistance in *C. albicans*.

The acetylation of Smc3 is essential for sister chromatid cohesion across various systems. Inactivation of the deacetylase Hdac8 in mammals (46) or depletion of Hos1 in budding yeast (47) results in increased levels of Smc3 acetylation, leading to delayed sister chromatid separation during mitosis. In *C. albicans*, Smc3 is acetylated at two adjacent sites, K104 and K105, which were found to be highly acetylated in four strains compared to Ca1. These conserved lysine residues K105/K106 (48) in humans, mice, rice, corn, and Arabidopsis; and K112/K113 (49) in budding yeast suggest that the dual acetylation of Smc3 may play a highly conserved role in regulating cell mitosis. Whole-chromosome LOH is a result of chromosome nondisjunction, which often leads to aneuploidy (50). Aneuploidy, characterized by the loss or gain of chromosomes, can enhance survival under stress conditions and is considered one of the mechanisms of resistance to FLC (51). During the *in vivo* acquisition of resistance, the acetylation levels of Smc3 significantly increase in parental *C. albicans* after FLC treatment. However, this increase diminished over time, suggesting that Smc3 acetylation may play a more prominent role in the early stages of acquired resistance in *C. albicans*.

We observed that the acetylation levels of two sites, K398 and K26, in the ribonucleotide reductase subunit Rnr21 were decreased in strains Ca2, Ca8, Ca14, and Ca17. Protein sequence alignment revealed that *C. albicans* Rnr21 shares high homology (72%) with human Rrm2. A previous study showed that acetylation at K95 in human Rrm2 mediates the inactivation of ribonucleotide reductase, leading to DNA replication fork stalling and inhibition of tumor growth (52). However, in this study, the homologous K115 acetylation site in *C. albicans* Rnr21 (the K95Ac site in human Rrm2) was not detected. Instead, two other distinct acetylation sites in Rnr21 exhibited significantly decreased acetylation levels. The impact of reduced acetylation at these two acetylation sites on the growth of *C. albicans* requires further confirmation in future studies.

The nucleosome is the fundamental structural unit of chromatin in eukaryotic organisms, consisting of an octamer of histones wrapped around a 147 bp DNA strand, including two copies each of the histones H2A, H2B, H3, and H4. We observed that acetylation levels in histones and associated proteins vary during the development of resistance in *C. albicans*. For example, Chz1, a yeast-specific partner protein of H2A.Z, exhibited decreased acetylation levels at the K123 site in strains Ca2, Ca8, Ca14, and Ca17. Chz1 is involved in the nuclear import of H2A.Z (53) and also plays a role in the transcription of subtelomeric genes (54), which may explain the increased expression of subtelomeric genes in the strains after FLC treatment. The protein encoded by orf19.7074, composed of the C-terminal domain of Sgf29—a component of the SAGA complex (Spt-Ada-Gcn5 acetyltransferase) (55)—showed decreased acetylation levels at the K121, K116, and K118 sites in the strains following FLC exposure. Sgf29 recruits the SAGA complex to its target sites by recognizing methylated H3K4 and mediating histone H3 acetylation (56). Although Sgf29 is not essential for the integrity of the SAGA complex, the absence of *SGF29* results in a decrease in the overall acetylation level of histone H3 (57), underscoring its significant role in gene regulation.

Regarding histones, H3K56ac in *C. albicans* is regulated by the fungus-specific acetyltransferase Rtt109 and deacetylase Hst3. Studies have shown that *rtt109Δ/Δ* strain, with decreased H3K56ac levels, is highly sensitive to the echinocandins (caspofungin and micafungin), possibly due to echinocandin-induced oxidative stress, which can be restored by antioxidants (58). Here, the acetylation level of H3K56 is also increased in Ca2 and Ca8, possibly correlating with the observed downregulation of oxidative stress levels in these strains from the proteomic analysis (25). Interestingly, H3K79ac, which is expressed at a low level in *Saccharomyces cerevisiae*, was over 10 times highly expressed in the Ca1 strain but decreased in the resistant strains in this study. Whether intervention in this relatively specific expression pattern of *C. albicans* can affect or reverse drug resistance warrants further investigation.

In summary, this study utilized a combined approach of proteomics, lysine acetylation, and PRM to initially screen for potential differential KAc sites associated with the development of azole antifungal resistance. This integrative analysis highlights the complex regulatory network of acetylation in azole resistance, indicating that it primarily offers metabolic and epigenetic support rather than directly driving core resistance targets. Although acetylation is not directly involved in classical resistance pathways, its role in global metabolism and epigenetic regulation suggests that targeting acetylation dynamics could be a potential strategy to weaken fungal resistance.

However, the limited sample size in this study may affect the comprehensiveness of the data. Therefore, future research will focus on incorporating a larger cohort of clinical isolates and developing point mutation models to identify and validate potential therapeutic targets.

## Data Availability

The mass spectrometry proteomics data have been deposited to the ProteomeXchange Consortium via the PRIDE (59) partner repository with the data set identifier PXDO57826.

## SUPPLEMENTAL MATERIAL

Data set S1 spectrum.02797-24-s0001.xlsx Three biological repetitions and integrated omics data.

Data set S2 spectrum.02797-24-s0002.xlsx DAPs in four groups and summary.

Supplemental figures spectrum.02797-24-s0003.docx Fig. S1 to S7.

Supplemental material spectrum.02797-24-s0004.docx Legends for Data set S1 and S2.

Table S1 spectrum.02797-24-s0005.xlsx Expression levels of different 155 KAc sites in four comparison groups.

Table S2 spectrum.02797-24-s0006.xlsx Quantitative information on 1171 KAc sites within 12 dynamic clusters.

ASM does not own the copyrights to Supplemental Material that may be linked to, or accessed through, an article. The authors have granted ASM a non-exclusive, world-wide license to publish the Supplemental Material files. Please contact the corresponding author directly for reuse.
