# Supplementary material for: Potential role of lysine acetylation in the stepwise adaptation of Candida albicans to fluconazole
Source: Microbiol Spectr. 2025 Apr 15;13(5):e02797-24. doi: 10.1128/spectrum.02797-24 (PMC12054006; doi:10.1128/spectrum.02797-24)
Supplement: Supplemental figures — Fig. S1 to S7. [file spectrum.02797-24-s0003.docx]

**Supporting Information**

**Potential Role of Lysine Acetylation in the Stepwise Adaptation of *Candida albicans* to Fluconazole**

**The Supporting Information includes supplemental figures as following:**

**Fig. S1** Motif analysis of acetylated proteins. **A**, A total of 16 motifs were identified to be significantly enriched at KAc sites. **B**, Enrichment heatmap of upstream and downstream amino acid motifs surrounding the identified KAc sites. Red and green indicate significant enrichment or significant reduction of these amino acids near the modification sites, respectively.

**Fig. S2.** GO-based enrichment analysis of significantly increased (red) and decreased (green) KAc proteins. **A** and **B**, group Q1 (Ca2 vs. Ca1).

**Fig. S3.** GO-based enrichment analysis of significantly increased (red) and decreased (green) KAc proteins. **A** and **B**, group Q2 (Ca8 vs. Ca1).

**Fig. S4.** GO-based enrichment analysis of significantly increased (red) and decreased (green, none) KAc proteins in group Q3 (Ca14 vs. Ca1).

**Fig. S5.** GO-based enrichment analysis of significantly increased (red) and decreased (green) KAc proteins. **A** and **B**, group Q4 (Ca17 vs. Ca1).

**Fig. S6.** KEGG-based enrichment analysis of significantly increased (red) and decreased (green) KAc proteins. **A** and **B**, Q1 (Ca2 vs. Ca1); **C** and **D**, Q2 (Ca8 vs. Ca1); **E**, Q3 (Ca14 vs. Ca1); **F**, Q4 (Ca17 vs. Ca1).

**Fig. S7.** Protein domain-based enrichment analysis of significantly increased (red) and decreased (green) KAc proteins. **A** and **B**, Q1 (Ca2 vs. Ca1); **C** and **D**, Q2 (Ca8 vs. Ca1); **E**, Q3 (Ca14 vs. Ca1); **F** and **G**, Q4 (Ca17 vs. Ca1).

**A**

**B**


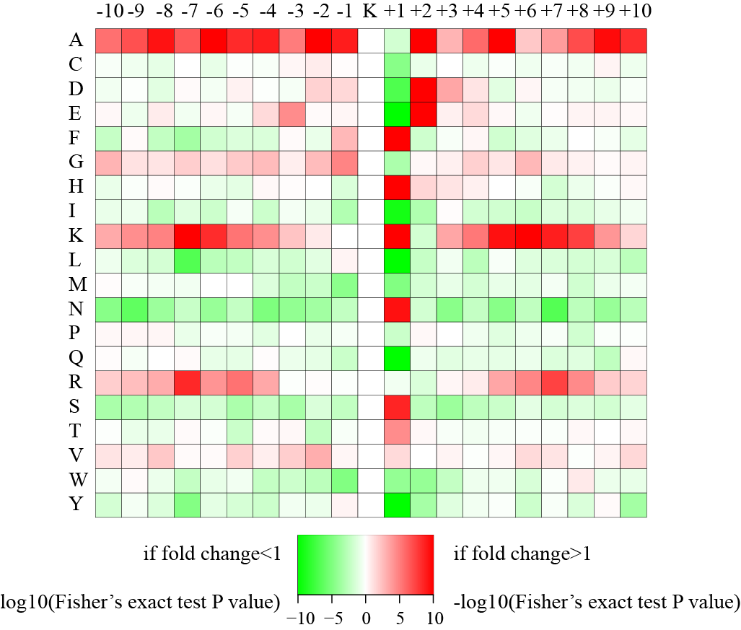


**Fig. S1** Motif analysis of acetylated proteins.

**A**


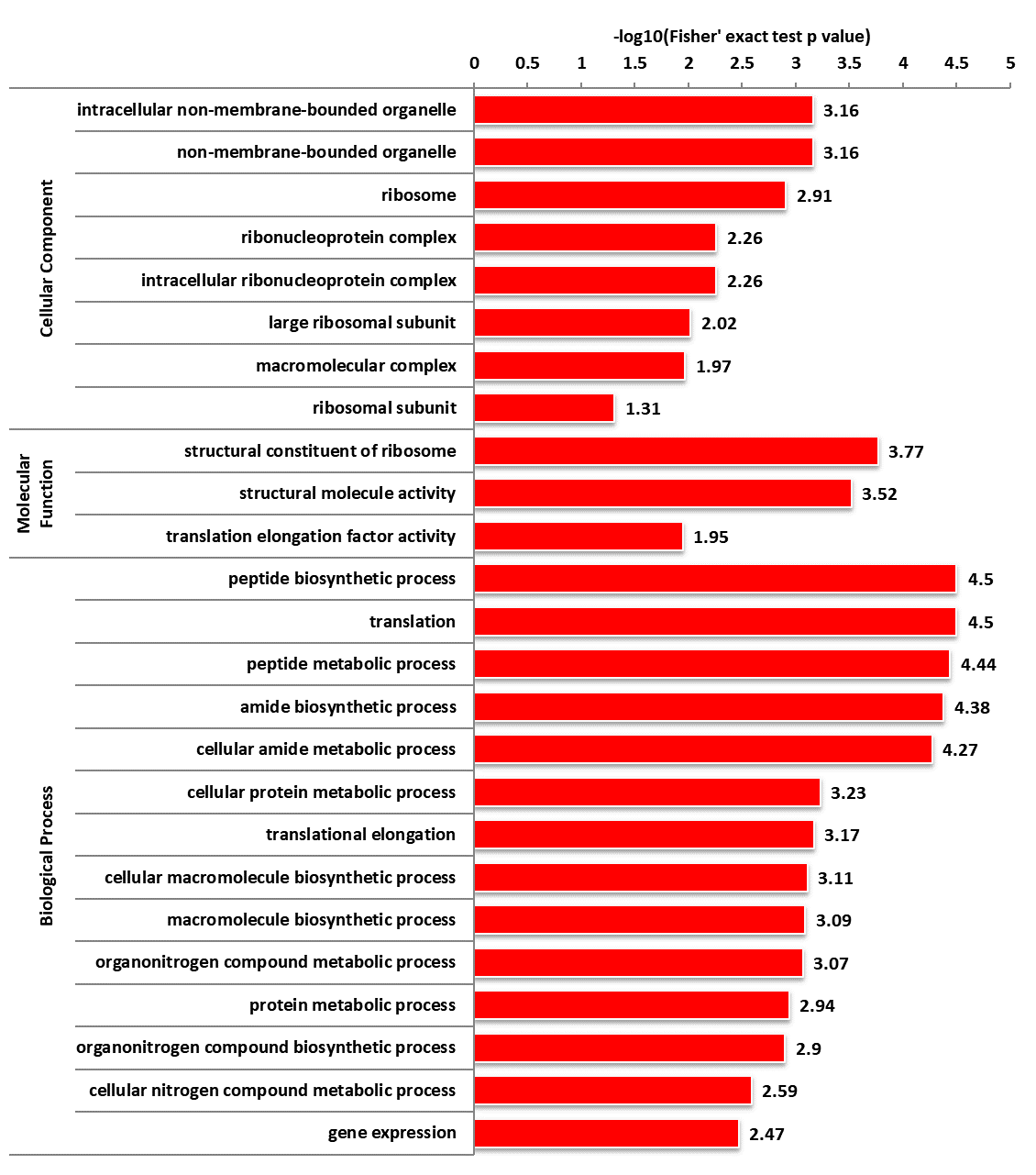


**B**


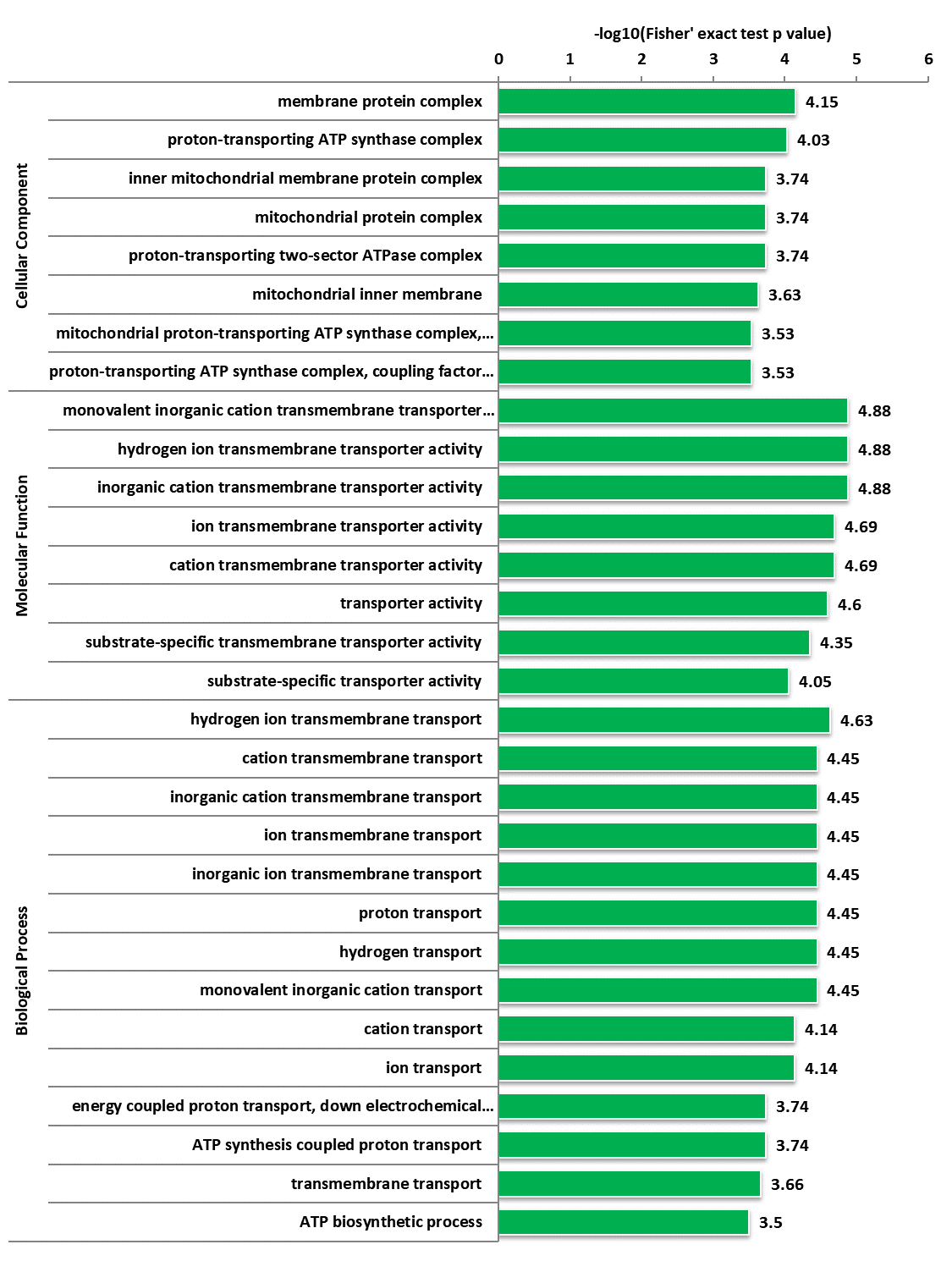


**Fig. S2.** GO-based enrichment analysis of significantly increased (red) and decreased (green) KAc proteins. **A** and **B**, group Q1 (Ca2 vs. Ca1).

**A**

**
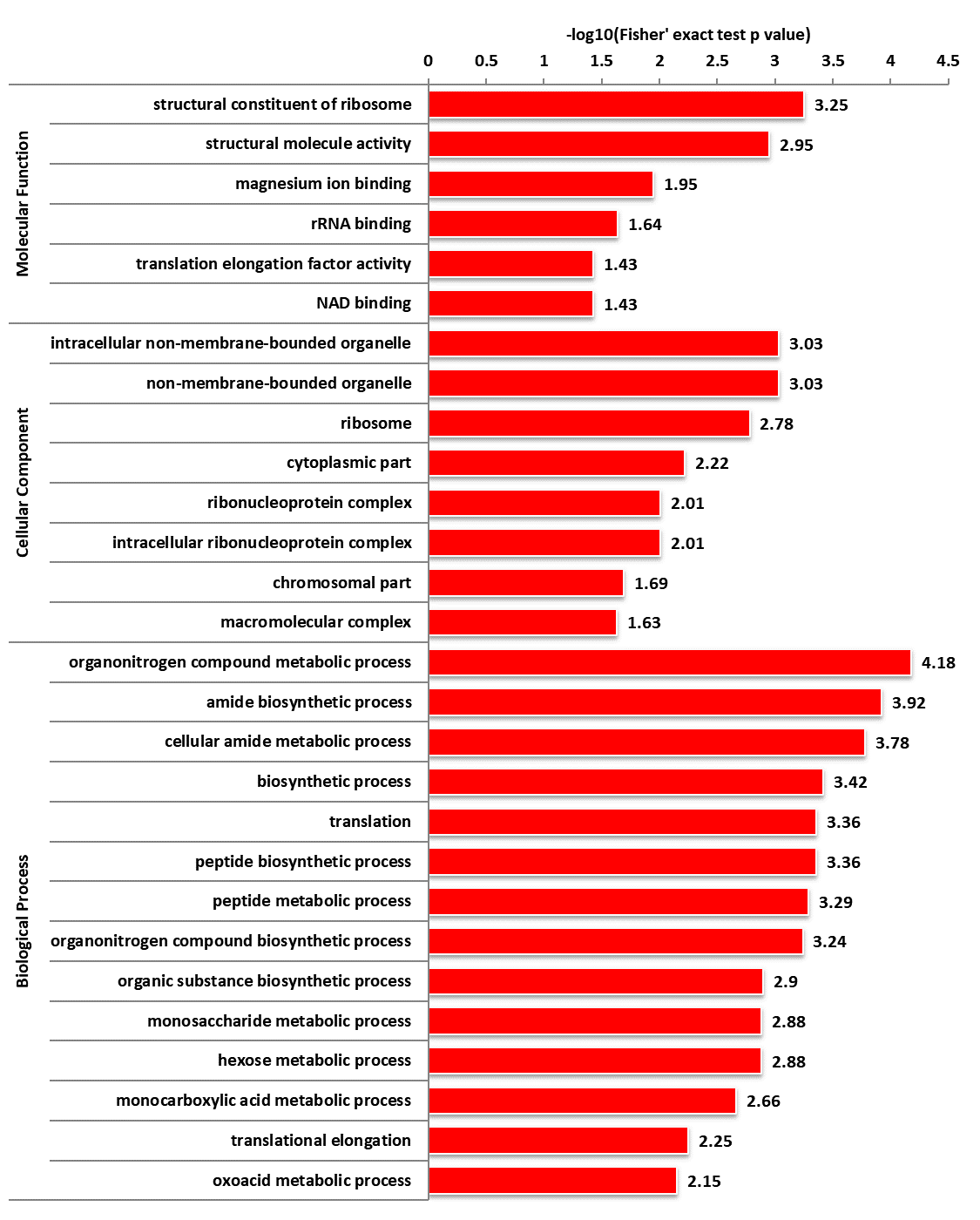
**

**B**

**
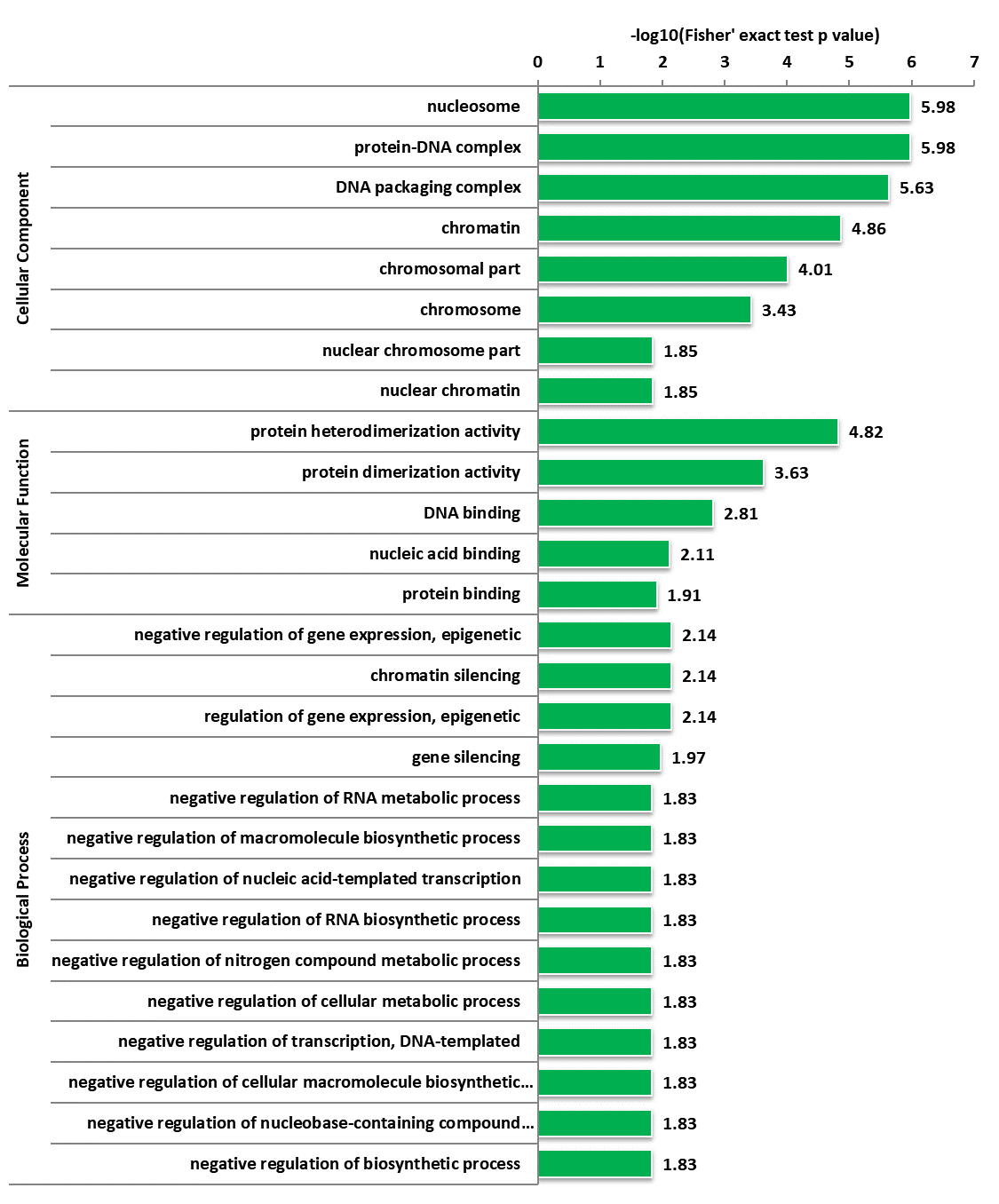
**

**Fig. S3.** GO-based enrichment analysis of significantly increased (red) and decreased (green) KAc proteins. **A** and **B**, group Q2 (Ca8 vs. Ca1).

**
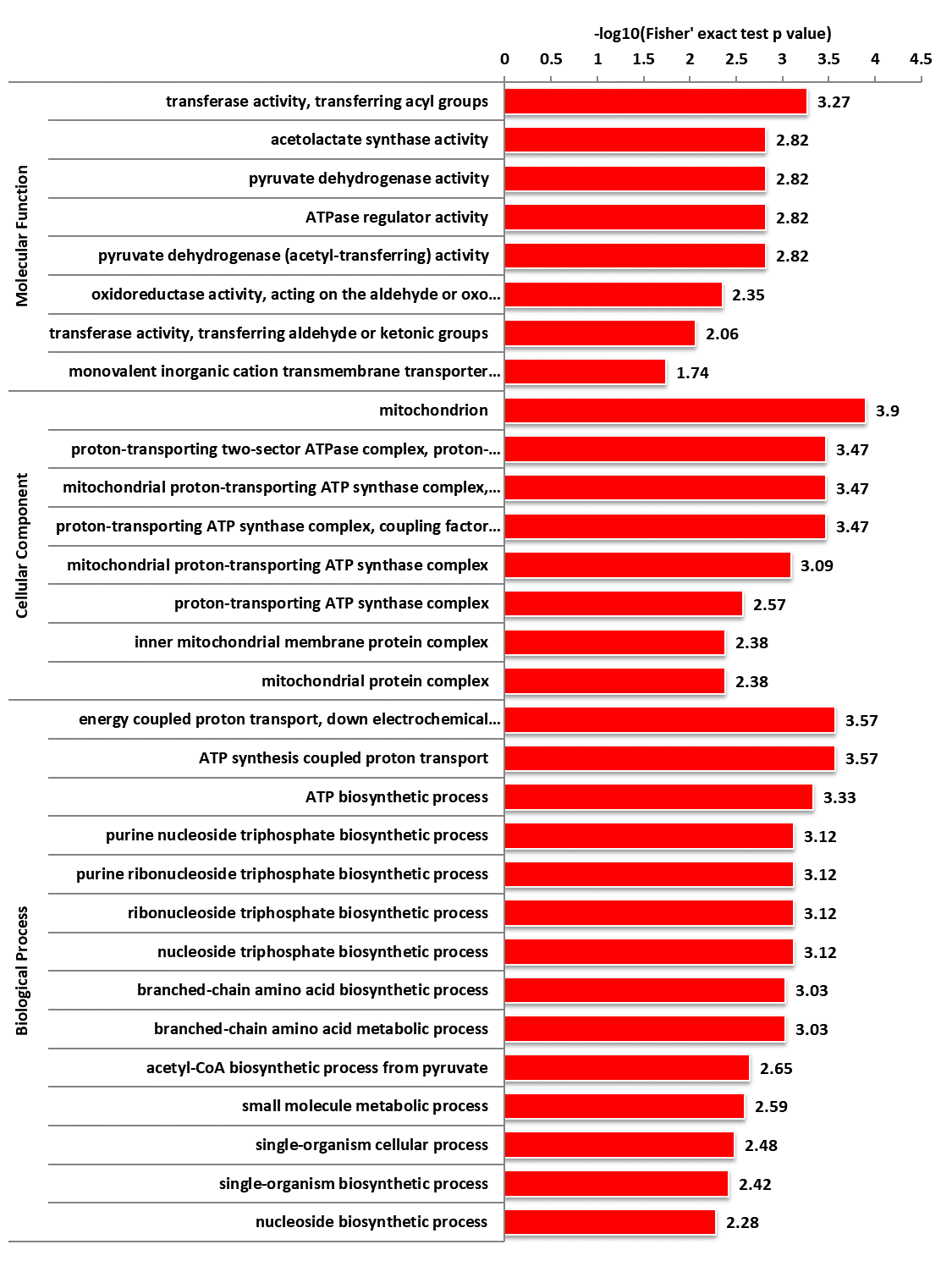
**

**Fig. S4.** GO-based enrichment analysis of significantly increased (red) and decreased (green, none) KAc proteins in group Q3 (Ca14 vs. Ca1).

**A**

**
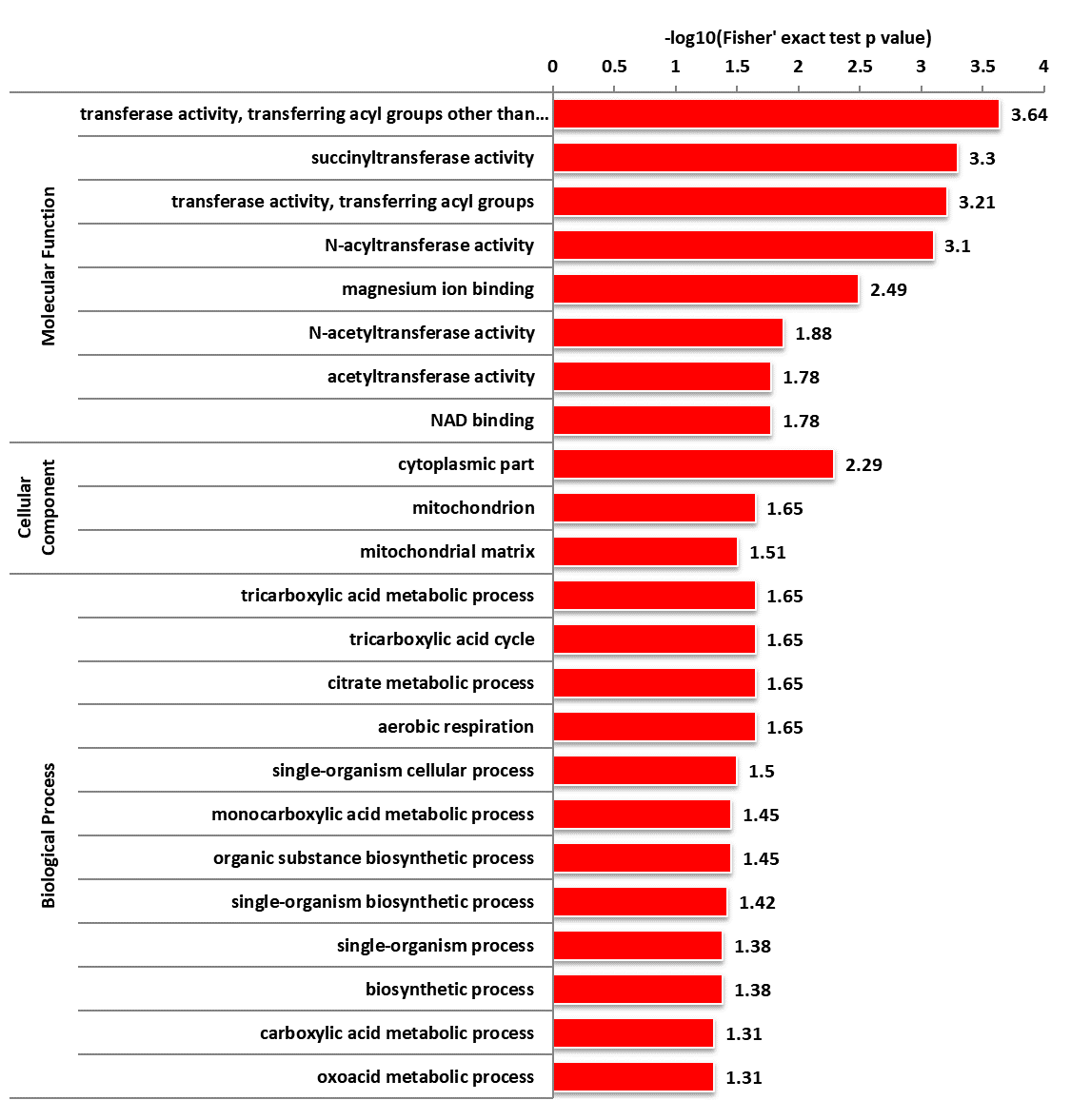
**

**B**

**
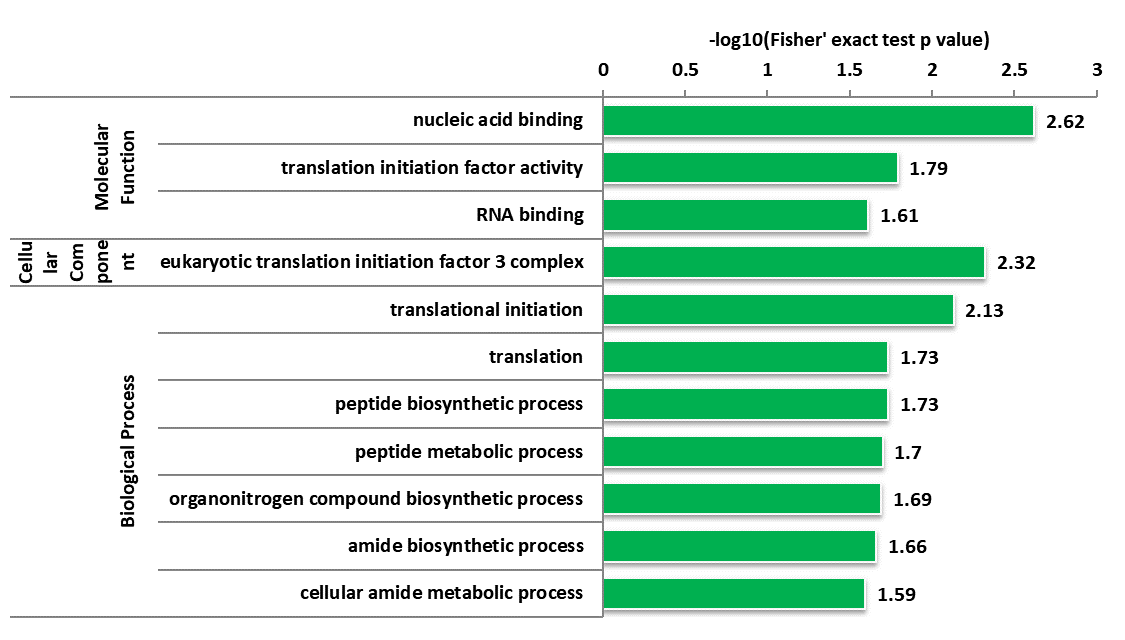
**

**Fig. S5.** GO-based enrichment analysis of significantly increased (red) and decreased (green) KAc proteins. **A** and **B**, group Q4 (Ca17 vs. Ca1).

**A**


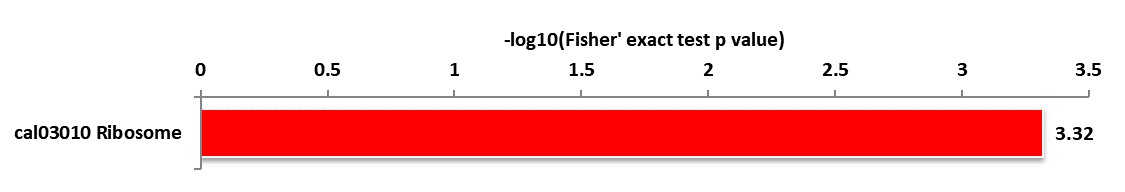


**B**


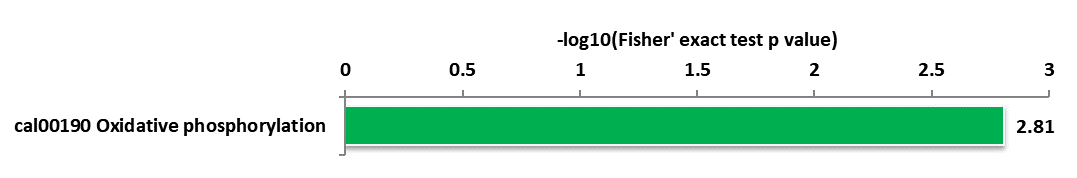


**C**

**
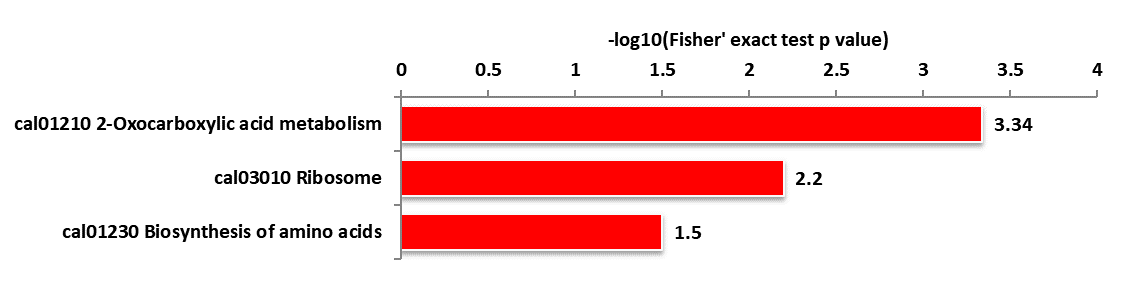
**

**D**

**
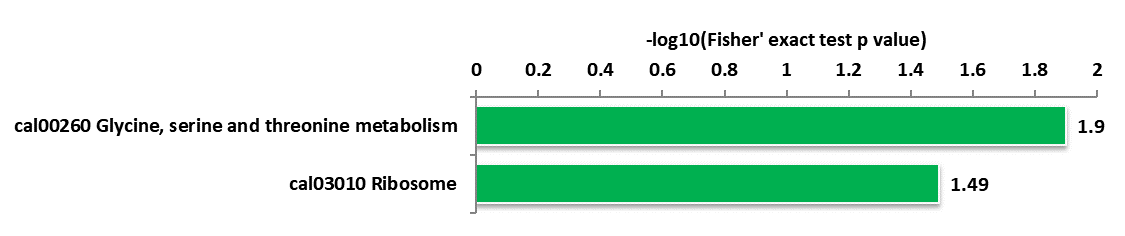
**

**E**

**
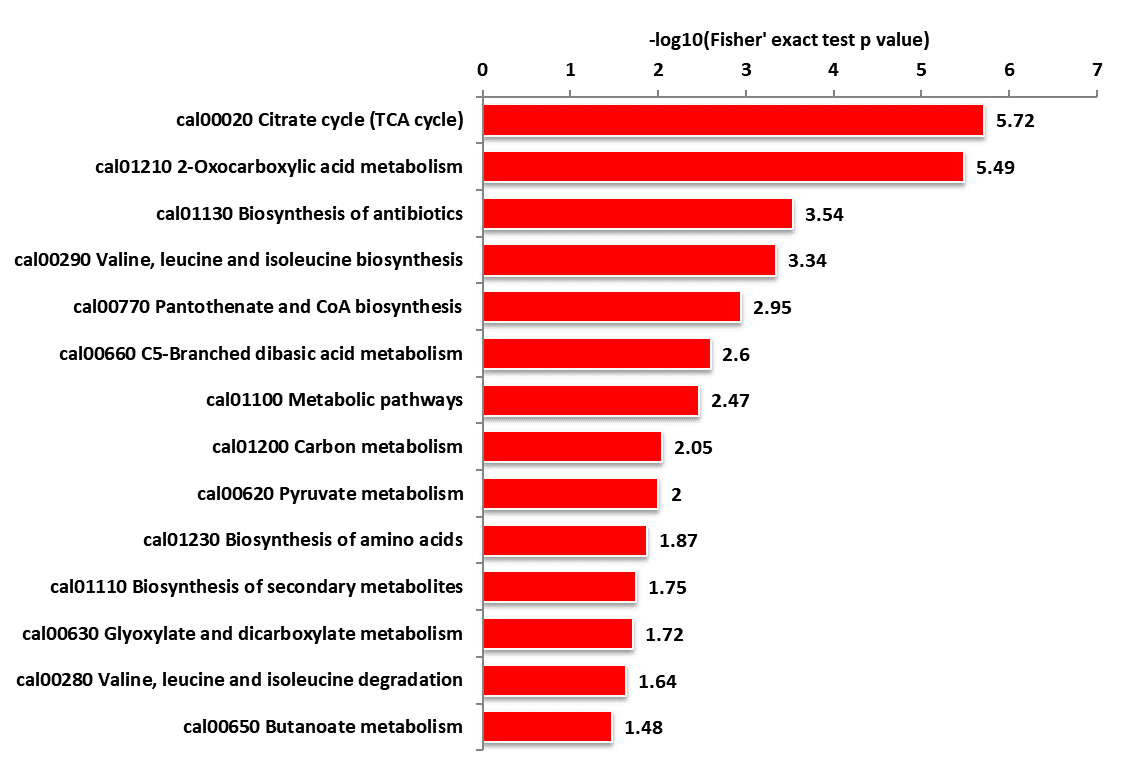
**

**F**

**
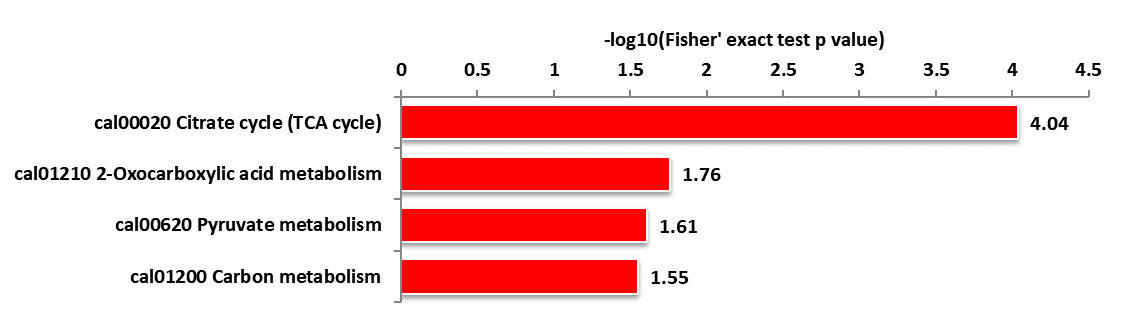
**

**Fig. S6.** KEGG-based enrichment analysis of significantly increased (red) and decreased (green) KAc proteins. **A** and **B**, Q1 (Ca2 vs. Ca1); **C** and **D**, Q2 (Ca8 vs. Ca1); **E**, Q3 (Ca14 vs. Ca1); **F**, Q4 (Ca17 vs. Ca1).

**A**


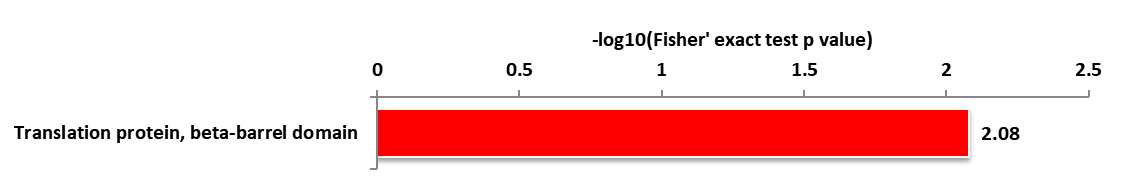


**B**

**
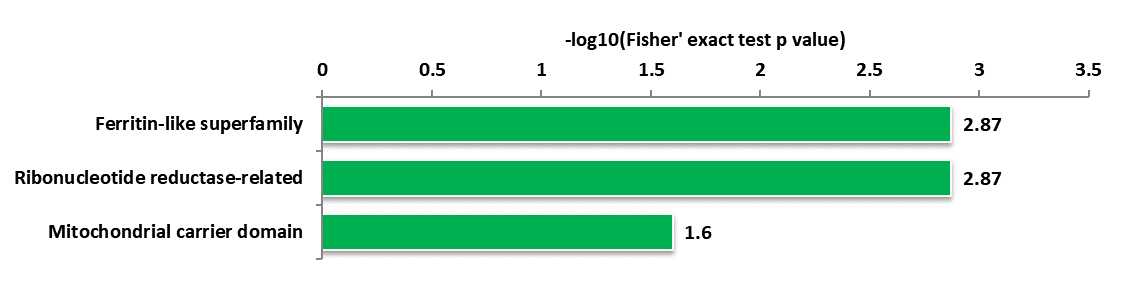
**

**C**

**
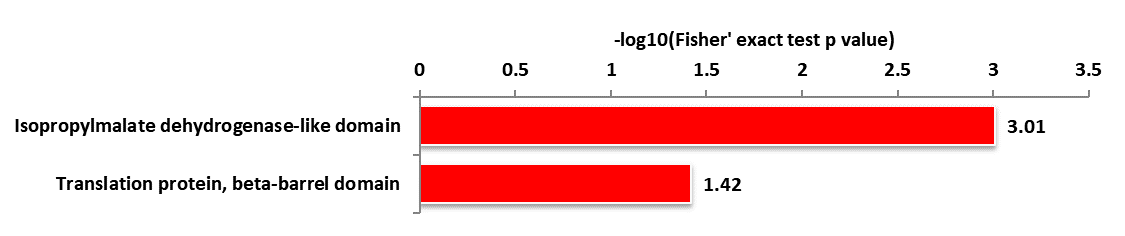
**

**D**

**
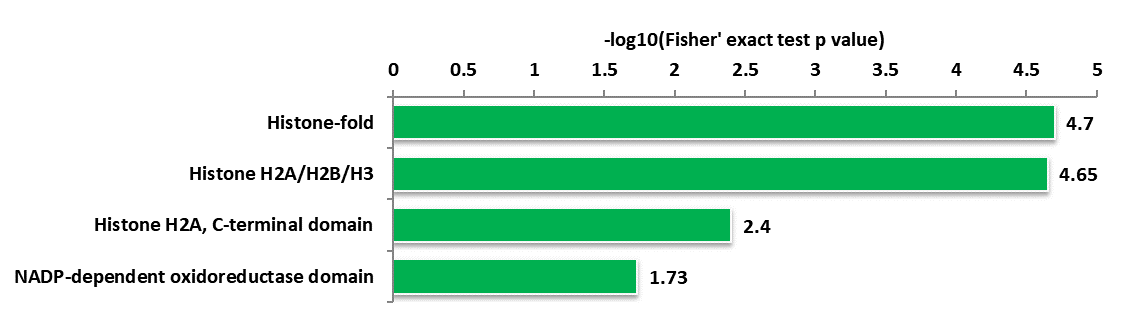
**

**E**

**
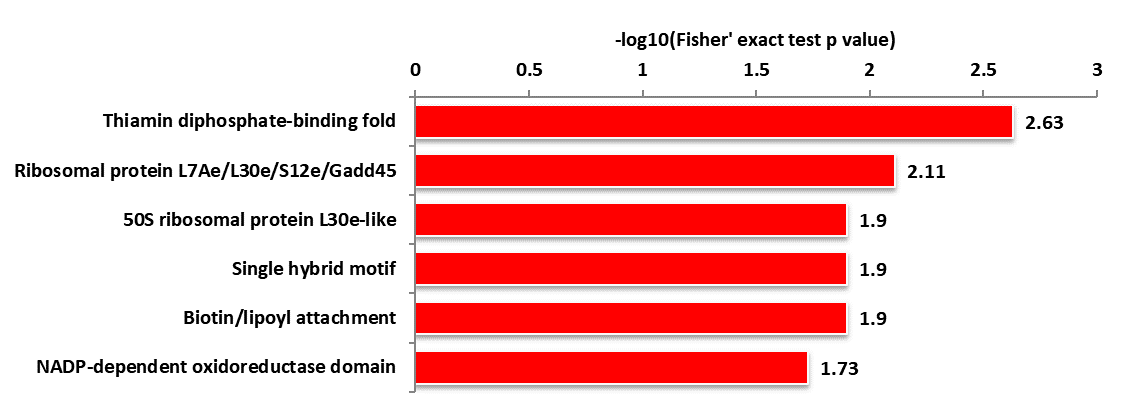
**

**F**

**
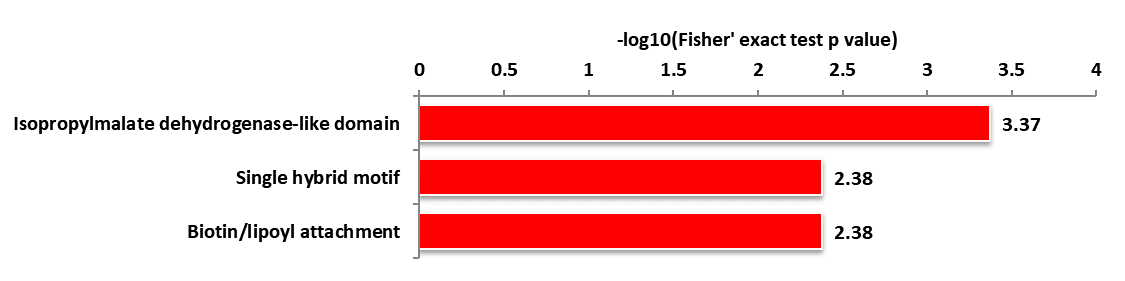
**

**G**

**
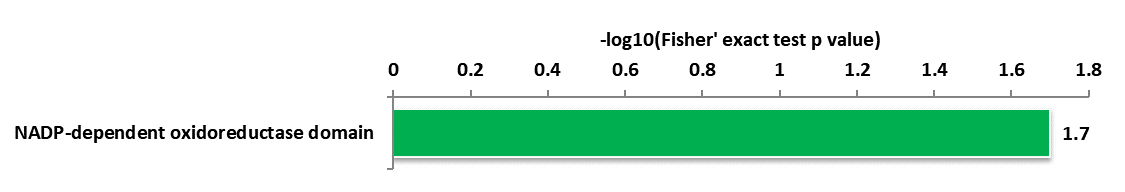
**

**Fig. S7.** Protein domain-based enrichment analysis of significantly increased (red) and decreased (green) KAc proteins. **A** and **B**, Q1 (Ca2 vs. Ca1); **C** and **D**, Q2 (Ca8 vs. Ca1); **E**, Q3 (Ca14 vs. Ca1); **F** and **G**, Q4 (Ca17 vs. Ca1).
