# Supplementary material for: Potential role of lysine acetylation in the stepwise adaptation of Candida albicans to fluconazole
Source: Microbiol Spectr. 2025 Apr 15;13(5):e02797-24. doi: 10.1128/spectrum.02797-24 (PMC12054006; doi:10.1128/spectrum.02797-24)
Supplement: Supplemental material — Legends for Data set S1 and S2. [file spectrum.02797-24-s0004.docx]

Legends for **Dataset S1** (Three Biological Repetitions and Integrated Omics Data)

This dataset contains 8 groups of data, which are arranged from left to right as follows (already named in the Excel sheet of the dataset):

1. **Summary of Table Header**: This part explains the abbreviations in some of the table headers.
2. **1 - Biological Repetition a**: It refers to the data from the first experiment of biological samples.
3. **2 - Biological Repetition b**: This is the data from the second biological replicate experiment under the same conditions.
4. **3 - Biological Repetition c**: It represents the data from the third biological replicate experiment under the same conditions.
5. **Repeatability Integration**: We integrate the above three sets of biological - repetition data in order to evaluate the repeatability and consistency of the experiment.
6. **Quantitative Proteome Data**: This is the proteome data obtained through quantitative analysis.
7. **Proteome - Acetylome Combination**: It includes basic information, acetylation information, proteome information, normalized acetylation information, combination information of the three - repetition - normalized data.
8. **Annotation of Combined Data**: It contains all the annotation information of the acetylome data after normalization.

Legends for **Dataset S2** (DAPs in Four Groups and Summary)

This dataset contains 5 groups of data, arranged from left to right as follows (already named in the Excel sheet of the dataset):

1. **Ca2 vs. Ca1**: This is the information on the differentially expressed lysine - acetylated (KAc) proteins in the comparison between group Ca2 and Ca1.
2. **Ca8 vs. Ca1**: It represents the information on the differentially expressed lysine - acetylated (KAc) proteins in the comparison between group Ca8 and Ca1.
3. **Ca14 vs. Ca1**: This is the information on the differentially expressed lysine - acetylated (KAc) proteins in the comparison between group Ca14 and Ca1.
4. **Ca17 vs. Ca1**: It refers to the information on the differentially expressed lysine - acetylated (KAc) proteins in the comparison between group Ca17 and Ca1.
5. **DAPs Summary**: This part summarizes the information on the differentially expressed lysine - acetylated (KAc) proteins in the above four groups.
